# Tracheotomy for Croup

**Published:** 1899-11

**Authors:** Charles A. Ellis

**Affiliations:** Sherman, N. Y.


### Clinical Report.

### TRACHEOTOMY FOR CROUP.

By CHARLES A. ELLIS, M. D., Sherman, N. Y.

I WAS summoned, October 28, 1896, to see a boy 12 years old who had taken cold and was hoarse. He was a thick-set, rugged, hardworking lad, who had been at the "tail end" of a threshing machine the day before in a cold, raw wind. His temperature was 101° F, pulse 90, had a hoarse cough, to which he was said to be subject; appetite good, bowels regular, no soreness of the throat, and he did not seem much ill. I left a stimulating expectorant and some aconite, requesting to be notified in the morning if he was not relieved.

At nine o'clock same evening I was hastily summoned, the messenger requesting me to be expeditious, as the boy seemed in extreme danger. On my arrival, some twenty minutes later, I found him gasping for breath, in a perspiration that had drenched his clothing. His pulse was rapid and his face bore an anxious, livid expression. An emetic was administered at once, but without relief. Explaining to his parents the need of intubation, I prepared to send for help and instruments, meanwhile hot applications to the throat were employed. In seven minutes from the time I arrived he stopped breathing.

His parents were informed that an incision would have to be made into the trachea to save his life, to which they consented. I made an incision into the trachea with a scalpel, using button hooks as retractors, and linen cloths, wrung out of a solution of permanganate of potash, for sponges. This was all the armamentarium that I could improvise. The incision through the trachea was soon accomplished, but so unconscious was the lad he never realised what was being done. Artificial respiration was begun, which soon developed regular, natural breathing. During this time I had sent to town for my emergency case and for Dr. C. H. Waterhouse. The distance was two miles and the boy was unconscious when he returned with some of the needed articles. Having no trachea tube we inserted strong silk strands into the edges of the trachea, and tied them behind his neck, but this did not seem satisfactory, so a thermometer case was cut off, heated in a lamp flame, bent into the desired shape, and used until a suitable one was supplied by Dr. C. B. Kibler, of Corry, Pa.

During these manipulations the patient became conscious and expressed himself as comfortable. His temperature fell to normal and he was feeling good the next day. His condition continued favorable and it seemed he would soon recover. On the morning of October 31st, I was again hastily summoned, as he was choking. On removing the tube it was found free from obstruction, but the difficult breathing continued. A probe touched an obstructing membrane, just below the position of the tube.

After the administration of a hypodermic of whiskey and 1-50 grain strychnine the patient revived somewhat, but was fast becoming exhausted. The case seemed hopeless, and I so informed the friends. Taking from my case a uterine curette that was very flexible, I bent it into a very satisfactory shape and putting it well down along the posterior wall of the trachea it passed the obstruction and with a quick backward motion it removed a membrane as I withdrew the curette. This membrane was an oblong "honey combed" mass which completely filled the lumen of the trachea for 2¼ inches by measure.

This procedure had to be repeated twice that day and once the next, but from that time forward it never returned. There was some sloughing around the incision, but not extensive. I at once gave a full injection of antitoxin. At this time his temperature was 104°, pulse rapid and he was very weak.

I gave him strychnia and a nutritious diet. He passed on to convalescence and soon became rugged and well as any lad of his years. He has had no return of his former attacks of croup. The pitch of his voice was the same for four months, since which time it has returned to normal. The house was quarantined and none of the other children contracted the disease.

